# A mediation analysis of the role of girl child marriage in the relationship between proximity to conflict and past-year intimate partner violence in post-conflict Sri Lanka

**DOI:** 10.1186/s13031-022-00436-2

**Published:** 2022-02-14

**Authors:** Ruvani W. Fonseka, Lotus McDougal, Anita Raj, Elizabeth Reed, Rebecka Lundgren, Lianne Urada, Jay G. Silverman

**Affiliations:** 1 Center on Gender Equity and Health (GEH), University of California San Diego School of Medicine, La Jolla, CA USA; 2 Joint Doctoral Program in Public Health, San Diego State University and University of California San Diego, San Diego, CA USA; 3 Present Address: School of Social Work, San José State University, San Jose, CA USA; 4 School of Public Health, San Diego State University, San Diego, CA USA; 5 School of Social Work, San Diego State University, San Diego, CA USA

**Keywords:** Sri Lanka, Intimate partner violence, Post-conflict setting, Girl child marriage, South Asia, Gender-based violence, Mediation analysis, Demographic and Health Survey (DHS)

## Abstract

**Background:**

Studies from many contexts indicate that proximity to conflict is associated with increased likelihood of intimate partner violence (IPV), and girl child marriage is associated with both proximity to conflict and increased IPV. In this study, we consider whether girl child marriage acts as a mediator of the association between proximity to conflict and IPV in the context of Sri Lanka, which sustained long-term conflict until 2009.

**Methods:**

We analyzed responses of currently partnered women between ages 18 and 49 in the 2016 Sri Lankan Demographic and Health Survey (N = 13,691). Using logistic regression analyses, we measured associations between proximity to conflict (residence in districts which were central, proximal, or distal to the regions where the war occurred) and the outcomes of IPV and girl child marriage, and secondarily assessed girl child marriage as a possible mediator of the association between proximity to conflict and past year IPV.

**Results:**

Women residing in districts central to conflict, as compared to districts distal to conflict, had increased odds of past year sexual, physical, and emotional IPV, with the odds of sexual IPV increasing the most (adjusted odds ratio/aOR 4.19, 95% confidence interval/CI 2.08–8.41). Residing in districts proximal to conflict compared to those distal to conflict was associated with lower odds of past year physical and emotional IPV, with the greatest decrease in emotional IPV (aOR 0.31, CI 0.18–0.54). Girl child marriage was more likely in districts central to conflict as opposed to those distal to conflict (aOR 1.89, CI 1.22–2.93), and partially mediated the relationship between centrality to conflict and IPV.

**Conclusions:**

Our findings demonstrate that residing in districts central to conflict compared to those distal to conflict is associated with greater odds of IPV and girl child marriage in post-conflict Sri Lanka, with girl child marriage partially mediating the association between centrality to conflict and IPV. Residence in districts proximal to conflict appears protective against IPV. Future research should investigate what factors are responsible for decreased IPV in districts proximal to violence, and whether these factors can be reproduced to mitigate the increased prevalence of IPV in districts central to conflict.

**Supplementary Information:**

The online version contains supplementary material available at 10.1186/s13031-022-00436-2.

## Background

Intimate partner violence (IPV) is a pressing public health issue across the world [1]. Across South Asia, the prevalence of IPV is estimated to be 41% [2]. Within the South Asian region, the risk factors and prevalence of IPV have not been studied to the same extent in Sri Lanka as in other countries due to the recent 30-year civil war, which ended in 2009 [3]. Multiple province- and district-level surveys have estimated the prevalence of IPV in post-conflict Sri Lanka to range from 30 to 40%, similar to the regional average. [4–6].

One potential driver of IPV in post-conflict Sri Lanka that is distinct from many other South Asian countries is proximity to areas where recent armed conflict occurred. In post-conflict settings in Sub-Saharan Africa, residing in conflict-affected areas has been shown to be associated with increased risk of IPV [7] and to impact mental health. [8, 9] Some of the identified risk factors of increased IPV in these settings include alcohol use [10, 11], war-related traumas [12, 13], changing gender norms and roles [14], and economic stressors including poverty and unemployment [10, 14]. Over the course of Sri Lanka's 30-year civil war, portions of the Northern and Eastern provinces were claimed as a separate state by the Liberation Tigers of Tamil Eelam (LTTE) organization [3]. Residents of the Northern and Eastern provinces were exposed to tremendous amounts of military violence and collective trauma, by both separatist and government forces [15]. The 2016 Sri Lankan Demographic and Health Survey (DHS), administered seven years after the end of the war, highlighted past year IPV prevalence rates of over 50% in multiple districts central to the conflict areas, providing evidence of a possible association between conflict exposure and IPV [16]. This possible association has been further supported by unpublished analyses of DHS data which considered conflict exposure as a binary variable. [17].

Girl child marriage has been shown in multiple settings to be associated with increased likelihood of IPV over a woman's lifetime [18, 19], and to increase in communities that have experienced armed conflict [20–23]. The region of South Asia has the second-highest prevalence of girl child marriage in in the world (29%) [24], and girl child marriage has been found to be associated with increased likelihood of IPV in both India and Bangladesh [25, 26]. In Sri Lanka, the singulate mean age of marriage (SMAM, a standard measure used to compare age of marriage across populations [27]) for women has risen and dipped over time, rather than the steady increase seen in neighboring countries and among Sri Lankan men, suggesting that the age of marriage for Sri Lankan women and girls is responsive to cultural shocks [28]. The legal minimum age of marriage in Sri Lanka for almost all women and girls is 18 [29]. During the war, girl child marriage was reported to be widely practiced among communities in areas central to the conflict, with nearly one in three women (31%) in one study reporting having been married by age 18 [30], compared to a national prevalence in the 2016 DHS of fewer than one in nine women (11.6%) [16]. Qualitative research conducted during and after the war's end found that early marriage was considered to be a protective strategy used by families to prevent their children from being recruited as combatants or experiencing sexual violence related to the conflict and displacement [30, 31]. Although these early marriage practices were reported to have started during the war, they have continued in war-affected communities in post-conflict Sri Lanka [31]. New norms, such as increased practice of underage customary marriages, have led to younger girls being married extralegally [31]. Research documenting that girl child marriage is associated with later IPV [19], combined with evidence that girl child marriage increased in Sri Lanka as a result of the conflict, suggests that girl child marriage could serve as a mediating factor between exposure to conflict and the experience of recent IPV.

This study aimed to understand the relationship between proximity to conflict and recent IPV experience in post-conflict Sri Lanka, and to test whether girl child marriage mediates this relationship. Analyzing the 2016 Demographic and Health Survey (DHS) data from Sri Lanka, we tested four hypotheses: (1) women in districts central to conflict will have increased odds of past year sexual, physical, and emotional IPV compared to women in districts distal to conflict; (2) women in districts central to conflict will have increased odds of having married as a child compared to women in districts distal to conflict; (3) women who married as children will have increased odds of past year sexual, physical, and emotional IPV compared to women married as adults; and (4) having married as a child will mediate the associations between proximity to conflict and past year IPV experience. The results of this study will build understanding of the relationships between conflict, girl child marriage, and IPV in Sri Lanka. Such knowledge may inform policymakers' efforts to develop post-conflict interventions to prevent future violence.

## Methods

### Data source

This study used data from the 2016 Sri Lankan Demographic and Health Survey (DHS), which collected individual-level data on child and maternal health outcomes, domestic violence experience, reproductive health, and economic engagement and agency of women in Sri Lanka (N = 27,210 households, 18,510 women aged 15–49). The 2016 survey was the first DHS to be conducted among a nationally-representative sample of households in Sri Lanka—all previous DHS data collection occurred during the 30-year civil war and excluded portions of the Northern and Eastern provinces.

In addition to a general health survey administered to every eligible woman in each household, a domestic violence module was administered to one randomly selected woman in each household (n = 16,629). The 2016 DHS was the first to ask questions on experience of past year intimate partner violence (IPV) in Sri Lanka. Multiple peer-reviewed studies have investigated child health [32, 33], postnatal care [34], and household decision-making [35] using data from the 2016 DHS. However, to date, no peer-reviewed studies have been published using the IPV data gathered in the domestic violence module. Following the World Health Organization’s guidelines for the ethical collection of information on domestic violence, each module respondent was read an additional consent statement at the start of the module, informing her that the questions could be personal and reassuring her of the confidentiality of her responses, and the module was not implemented if privacy could not be obtained [36]. In the 2016 DHS, women taking part in the domestic violence module were asked nine questions about their experiences of intimate partner violence in the previous 12 months. [16] This study was restricted to women who were currently living with an intimate partner and who answered the domestic violence module of the DHS. This study was also restricted to women age 18 and above, following a convention in girl child marriage research to exclude girls still within the window of risk for girl child marriage [37]. This practice is followed because it is possible that unmarried girls might marry after survey data collection but before their 18^th^ birthday. The complete sample of women included in this study comprised of 13,691 participants. Ethical approval for this secondary analysis of de-identified data was obtained from the University of California, San Diego Institutional Review Board (Project number #191418XX).

### Variables of interest

#### Dependent variables: past year intimate partner violence (IPV) – sexual, physical, or emotional

We examined three different IPV variables as dependent variables of interest. The three binary variables are listed below:

1. Past year sexual IPV: having been forced to have sex by a partner in the last 12 months; yes or no.

2. Past year physical IPV: having experienced at least one of six types of physical IPV in the last 12 months: (1) slapping or beating with a hand, (2) pushing or shoving, (3) strangulation, (4) dragging or pulling, (5) beating with an object, or (6) burned; yes, or no.

3. Past year emotional IPV: having experienced at least one of two types of emotional IPV in the last 12 months: (1) being belittled/offended or (2) prevented from leaving home by a partner; yes or no.

In our analyses, we considered sexual, physical, and emotional IPV as separate outcomes, based on Spearman's rho correlation estimates across all three IPV variables remaining below 0.5.

#### Independent variable: proximity to conflict

**Fig. 1:**
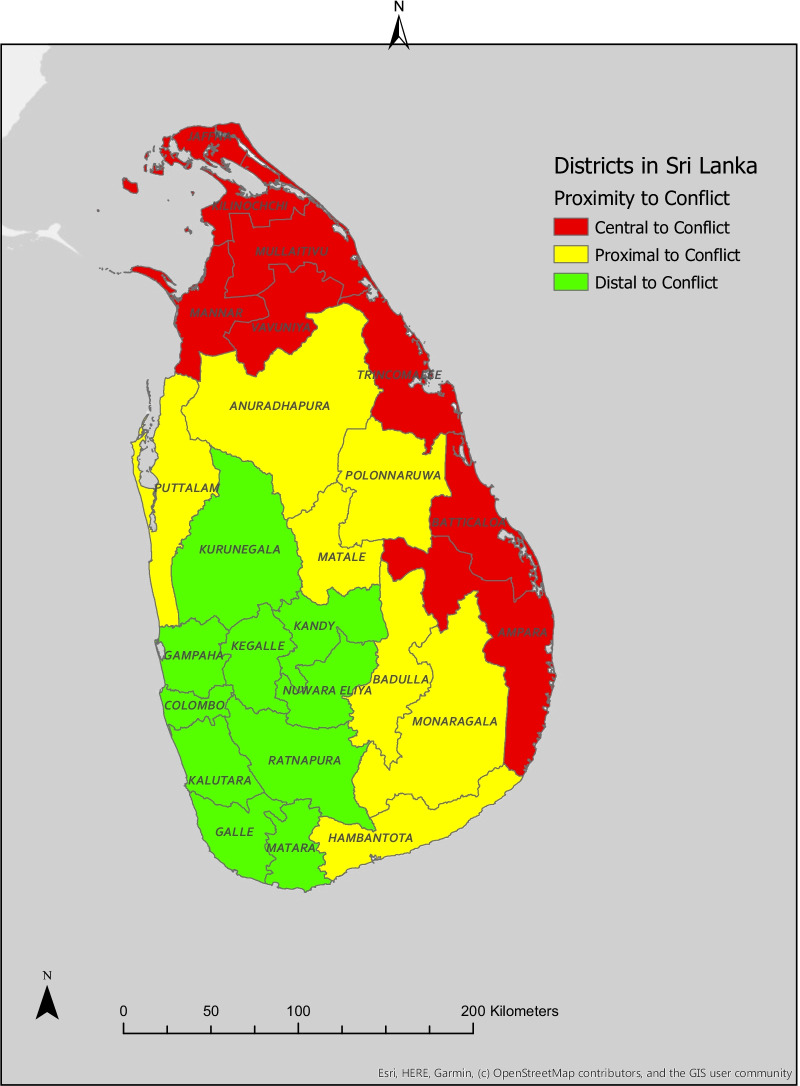
Map of Sri Lankan Districts. Adapted from Fonseka RW. Understanding gender-based violence and health in post-conflict Sri Lanka. University of California San Diego; 2021

We included proximity to conflict as the independent variable in our analyses. This variable was defined as having three ordinal levels: distal, proximal and central. Participants assigned to the distal category resided in districts that were neither in nor bordering the Northern and Eastern provinces of the country, where most of the armed conflict occurred during the civil war. Distal districts included Colombo, Galle, Gampaha, Kalutara, Kandy, Kegalle, Kurunegala, Matara, Nuwara Eliya, and Ratnapura. Participants assigned to the proximal category reported residing in one of the seven districts of Sri Lanka outside of but sharing a border with at least one district within the Northern and Eastern provinces. Proximal districts included Anuradhapura, Badulla, Hambantota, Matale, Monaragala, Polonnaruwa, and Puttalam. Finally, participants assigned to the central category reported residing in a district located within either the Northern or Eastern provinces of Sri Lanka. Central districts included Ampara, Batticaloa, Jaffna, Kilinochchi, Mannar, Mullaitivu, Trincomalee, and Vavuniya. A map of Sri Lanka showing the geographic distribution of proximity to conflict is included as Fig. 1.

### Potential mediator: girl child marriage

Informed by prior research conducted in Sri Lanka and other countries on conflict, IPV, and girl child marriage, we considered girl child marriage to be a potential mediator of the relationships between proximity to conflict and past year IPV. Girl child marriage was measured as a binary yes/no variable based on whether a woman had married or cohabited with a male partner before age 18.

### Covariates

We included as covariates in the relationship between proximity to conflict and past year IPV variables known or hypothesized to be associated with IPV. These variables included each respondent's age (in years), education (primary or less, secondary, or higher than secondary), household wealth quintile, parity (0–1, 2, or 3 or more), the age difference between a respondent and her partner (in years), religion (Buddhism, Hinduism, Islam, or other), and ethnicity (Sinhala, Sri Lankan Tamil, or other). Beyond individual-level characteristics, we also included as covariates whether the respondent lived in an urban setting or not, and which district in Sri Lanka she lived in, to control for unmeasured variation at the community level.

### Analysis

We first assessed the distributions of all three forms of past year IPV, proximity to conflict, girl child marriage, and all considered covariates in the sample. Next, we tested if the pairwise distributions of all the variables across past year IPV types were significantly different. We used chi-squared tests for all comparisons across past year IPV type after reducing all variables to categories (ordinal or nominal). In preparation for our multivariable model, we assessed all variables for multicollinearity by calculating their variance inflation factor (VIF); variables with VIF values above 5 were examined for overlap in distribution with other variables in the model. We kept in the analysis all covariates which could be retained with variance inflation factor (VIF) values less than 5, eliminating only ethnicity.

**Fig. 2:**
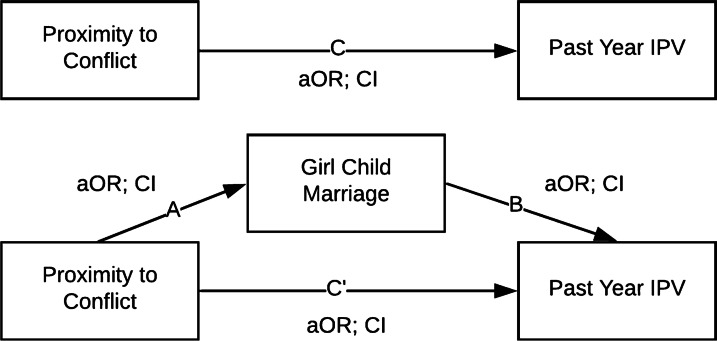
Measuring whether girl child marriage mediates the relationship between proximity to conflict and past-year intimate partner violence (IPV) in post-conflict Sri Lanka. Legend: aOR: adjusted odds ratio; CI: 95% confidence interval

After examining bivariable associations, we conducted a mediation analysis with a series of adjusted logistic regressions, following the four steps suggested by Baron and Kenny [38]. Mediation analysis is a statistical technique that allows researchers to identify possible intermediary pathways between an independent and dependent variable. In our case, based on the literature summarized in the introduction, we considered whether girl child marriage could be the intermediary mechanism linking proximity to conflict to experiencing IPV. The different paths we analyzed are labeled in Fig. 2.

First, we analyzed the relationships between proximity to conflict (the independent variable) and each form of past year IPV (the dependent variables) while excluding girl child marriage (the proposed mediator), modeling the C path in Fig. 2. Next, we analyzed the relationship between proximity to conflict and girl child marriage, excluding the past year IPV variable being considered as the dependent variable, modeling the A path in Fig. 2. Finally, we ran full adjusted logistic regression models with proximity to conflict and girl child marriage both included as predictors of each past year IPV variable (calculating the B and C' paths in Fig. 2). For a full mediating effect, the adjusted odds ratio (aOR) of proximity to conflict on past year IPV (C) should lose statistical significance (C' p-value greater than 0.05) after the mediator is included in the model. A partial mediating effect exists when the impact of proximity to conflict on past year IPV is reduced when girl child marriage is included in the model (the aOR for C' is closer to 1 than C). All statistical analyses were conducted using R software version 3.6.3 [39], and estimates were weighted using the "survey" package [40] in order to calculate population-representative measures that took into account the complex sampling design of the DHS.

## Results

### Descriptive statistics

Demographic characteristics of the sample and the distribution of past year IPV by characteristic are displayed in Table 1. The type of past-year IPV most prevalent among respondents was emotional IPV (13%), while past year sexual IPV had the lowest prevalence (2%). Over half of the participants lived in districts that were distal to conflict (64%) and had not been married as children (86%). Most women had obtained some secondary education (66%), practiced Buddhism (73%), and did not live in an urban setting (85%).

**Table 1:** Demographic details of currently partnered women aged 18–49 who participated in the 2016 Sri Lankan DHS Domestic Violence module and distribution of past year sexual, physical, and emotional intimate partner violence (IPV) (N = 13,691)

	Total		Past year sexual IPV			Past year physical IPV			Past year emotional IPV		
Characteristic	n	%^	n	%	Chi-squared *p* value	n	%	Chi-squared *p* value	n	%	Chi-squared *p* value
Total	13,691	100	363	2	–	1290	9	–	1949	13	–
Proximity to conflict											
Distal	7603	64	110	1	< 0.01*	529	7%	< 0.01*	867	11	< 0.01*
Proximal	3131	23	60	2		247	8		261	9	
Central	2957	13	193	8		514	19		821	29	
Girl child marriage											
No	11,707	86	265	2	< 0.01*	967	8	< 0.01*	1539	12	< 0.01*
Yes	1984	14	98	5		323	16		410	19	
Age^^											
18–29	3031	22	69	2	0.03*	276	8	0.04*	394	12	< 0.01*
30–39	5852	43	147	2		543	8		804	13	
40–49	4808	35	147	3		471	10		751	15	
Education											
Primary (01–05) or less	1189	8	76	6	< 0.01*	234	20	< 0.01*	288	24	< 0.01*
Secondary (6–12)	9119	66	252	2		919	10		1303	13	
Higher than Secondary	3383	26	35	1		137	4		358	10	
Household wealth quintile											
Lowest	3090	18	169	5	< 0.01*	575	19	< 0.01*	734	22	< 0.01*
Second	2787	20	89	3		276	10		399	14	
Middle	2683	21	42	2		200	7		305	11	
Fourth	2660	21	33	1		146	6		270	10	
Highest	2471	20	30	1		93	4		241	10	
Parity											
0–1	4267	32	62	1	< 0.01*	276	6	< 0.01*	475	10	< 0.01*
2	5154	39	123	2		470	9		687	12	
3+	4270	29	178	4		544	12		787	17	
Age difference between woman and partner^^											
Same or older	2468	18	77	3	0.12	283	11	< 0.01*	397	15	0.05*
1–5 years	6712	49	176	2		628	9		911	12	
6–10 years	3598	27	77	2		285	7		494	13	
Over 10 years	913	6	33	3		94	9		147	14	
Religion											
Buddhism	8855	73	135	2	< 0.01*	585	7	< 0.01*	922	11	< 0.01*
Hinduism	2327	11	127	6		417	19		624	27	
Islam	1267	9	59	4		132	10		195	15	
Other	1242	8	42	3		156	12		208	15	
Urban setting											
No	11,510	85	312	2	0.85	1097	9	0.56	1604	13	< 0.01*
Yes	2181	15	51	2		193	9		345	16	
District^^^					< 0.01*			< 0.01*			< 0.01*

n values are unweighted, while percent values are weighted

Chi-squared p-values are weighted according to the survey's complex sampling design

^Total percent values are calculated within the same column, while all other percent values are calculated across the same row

^^Age and age difference between woman and partner are presented in cross-tabulations as categorical but were included in regression models as continuous variables

^^^A full list of districts is omitted due to length (see Additional file 1)

^*^p < 0.05

### Bivariate analyses between IPV and other variables

Cross-tabulations of all forms of past year IPV and other variables are presented in Table 1. All forms of past year IPV were most prevalent among women in districts central to conflict and among women who were married as children. Almost all demographic characteristics were significantly associated (*p* < 0.05) with each form of IPV. All IPV variables were most prevalent among households with lower wealth, and among women with less education, higher parity, and who practiced Hinduism. Cross-tabulations of past year IPV with all districts in Sri Lanka can be found in Additional file 1.

### Regression analyses between proximity to conflict and IPV

In our regression models, we controlled for age, education, household wealth quintile, parity, age difference between woman and partner, religion, urban, setting, and district. All regression estimates can be found in Table 2. Not controlling for girl child marriage, women in districts central to conflict were significantly more likely to experience all three forms of past year IPV than women in distal districts, with the greatest increase in odds occurring for past year sexual IPV (aOR 4.19, 95% Confidence Interval/CI 2.08–8.41). Conversely, women in districts proximal to conflict were significantly less likely to experience past year physical and emotional IPV than women in distal districts, with the greatest reduction in odds occurring for past year emotional IPV (aOR 0.31, CI 0.18–0.54). There was not a significant difference in the odds of sexual IPV between women in districts proximal to conflict and women in distal districts (aOR: 0.34, CI 0.09–1.23).

**Table 2:** Logistic regression analyses of the relationship between proximity to conflict, girl child marriage, and past year sexual, physical, and emotional intimate partner violence (IPV) among currently partnered women aged 18–49 who participated in the 2016 Sri Lankan DHS Domestic Violence module (N = 13,691)

	C path (outcome: IPV = yes)			A path (outcome: Child Marriage = yes)			C' and B path (outcome: IPV = yes)		
Variable of interest	aOR	CI	*p* value	aOR	CI	*p* value	aOR	CI	*p* value
*Relationships between proximity to conflict, girl child marriage, and past year sexual IPV (SIPV)*									
Proximity to conflict									
Distal	ref	ref	ref	ref	ref	ref	ref	ref	ref
Proximal	0.34	0.09, 1.23	0.10	1.28	0.83, 1.97	0.30	–	–	–
Central	4.19	2.08, 8.41	< 0.01*	1.89	1.22, 2.93	< 0.01*	4.03	2.00, 8.12	< 0.01*
Girl child marriage									
No	–	–	–	–	–	–	ref	ref	ref
Yes	–	–	–	–	–	–	1.55	1.15, 2.09	< 0.01*
*Relationships between proximity to conflict, girl child marriage, and past year physical IPV (PIPV)*									
Proximity to conflict									
Distal	ref	ref	ref	ref	ref	ref	ref	ref	ref
Proximal	0.51	0.29, 0.91	0.02*	1.28	0.83, 1.97	0.30	–	–	–
Central	2.15	1.36, 3.41	< 0.01*	1.89	1.22, 2.93	< 0.01*	2.07	1.30, 3.30	< 0.01*
Girl child marriage									
No	–	–	–	–	–	–	ref	ref	ref
Yes	–	–	–	–	–	–	1.62	1.37, 1.93	< 0.01*
*Relationships between proximity to conflict, girl child marriage, and past year emotional IPV (EIPV)*									
Proximity to conflict									
Distal	ref	ref	ref	ref	ref	ref	ref	ref	ref
Proximal	0.31	0.18, 0.54	< 0.01*	1.28	0.83, 1.97	0.30	–	–	–
Central	1.88	1.30, 2.70	< 0.01*	1.89	1.22, 2.93	< 0.01*	1.82	1.26, 2.63	< 0.01*
Girl child marriage									
No	–	–	–	–	–	–	ref	ref	ref
Yes	–	–	–	–	–	–	1.45	1.23, 1.71	< 0.01*

Regression models included as covariates age, education, household wealth quintile, parity, age difference between woman and partner, religion, urban setting, and district

Confidence intervals and p-values are weighted according to the survey's complex sampling design

aOR: adjusted odds ratio; CI: 95% confidence interval

^*^: p < 0.05

### Mediation analyses of the role of girl child marriage

**Fig. 3:**
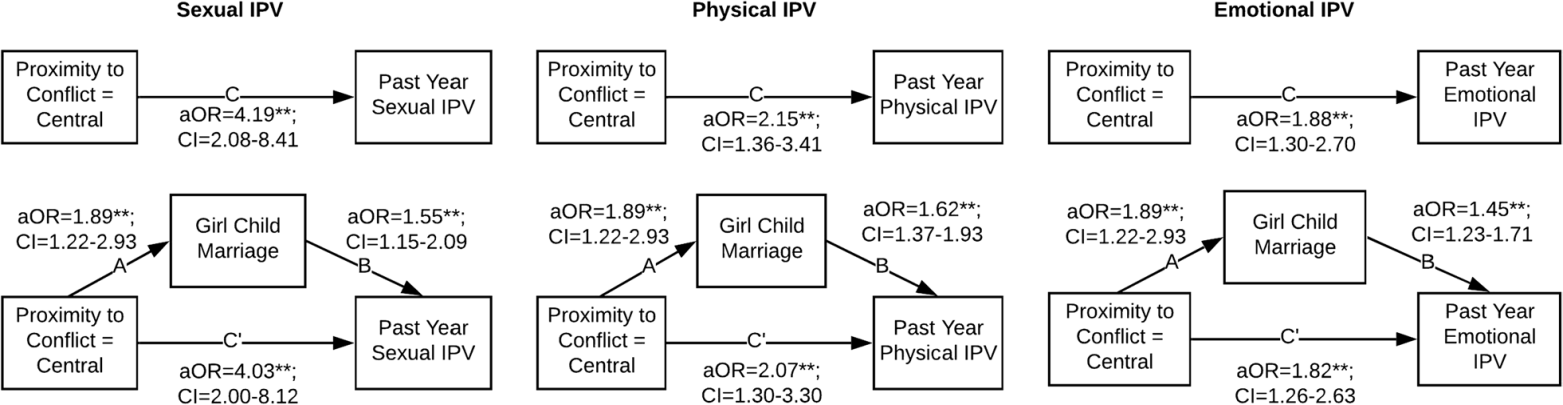
Measuring whether girl child marriage mediates the relationship between proximity to conflict (comparing central to distal) and past year sexual, physical, and emotional intimate partner violence (IPV) in post-conflict Sri Lanka. Legend: aOR: adjusted odds ratio; CI: 95% confidence interval; **:*p* < 0.01; odds ratios use proximity to conflict = distal as reference group

All mediation path estimates for past year sexual, physical, and emotional IPV are shown in Fig. 3. The only significant (*p* < 0.05) association between proximity to conflict and girl child marriage (the A path) was found when comparing districts that were central to conflict to distal districts (aOR 1.89, CI 1.22–2.93, Table 2). Therefore, we only investigated the role of girl child marriage as a mediator of proximity to conflict and IPV (the C' and B paths) among women in districts central to conflict compared to women in distal districts. We found that the associations between girl child marriage and each form of past year IPV (the B paths) were significant. The adjusted odds ratio of women experiencing each form of past year IPV in districts central to conflict compared to women in distal districts decreased with the addition of girl child marriage (comparing the C and C' paths) by less than 5 percent (sexual IPV: 4%, physical IPV: 4%, emotional IPV: 3%), suggesting partial mediation.

## Discussion

Our study provided evidence in support of our hypotheses about the relationships between proximity to conflict, IPV and girl child marriage in post-conflict Sri Lanka. Women in districts central to conflict had increased odds of all forms of past year IPV and girl child marriage compared to women in distal districts. Women who married as children had increased odds of past year sexual, physical, and emotional IPV compared to women who married as adults, and girl child marriage partially mediated the associations between proximity to conflict and past year IPV experience. These findings support and add to the established research on associations between proximity to conflict, IPV and girl child marriage in different settings.

Our study provides evidence from South Asia to add to the growing literature that links centrality to conflict with increased IPV experience in other post-conflict settings, mainly in Sub-Saharan Africa. We found that women in districts central to conflict in Sri Lanka had greater adjusted odds of recent IPV experience than women in distal districts. Qualitative research in post-conflict Uganda has also suggested associations between armed conflict and physical and sexual IPV [41], while quantitative research from post-conflict Liberia found that women living in conflict-affected areas were more likely to experience any past year IPV compared to women in other parts of the country [7]. One framework that might explain the impact on IPV of various explanatory factors, including conflict, is the ecological model adapted by Heise from Bronfenbrenner, which posits that the likelihood of IPV is influenced by many different dimensions of one's environment, from the interpersonal to the societal [42, 43]. This framework has been further adapted to explicitly consider the role of conflict and post-conflict factors by Swaine et al., who highlighted the role of conflict in normalizing violence and changing gender dynamics in affected communities [44]. These changing post-conflict gender dynamics have been shown in multiple settings to make violence against women and girls more likely [14, 45]. Our study adds to this literature to further support the idea that women living in conflict-affected areas of a post-conflict country are at increased risk of IPV compared to women living farther from the site of armed conflict.

Our study quantitatively confirms a positive association between centrality to conflict and girl child marriage. Additionally, it provides evidence that girl child marriage partially mediates the positive association between all forms of IPV and centrality to conflict in Sri Lanka. These findings support qualitative research done in Sri Lanka on the links between conflict and girl child marriage as well as similar quantitative studies in other conflict-affected settings of associations between girl child marriage and IPV. A previous study of women and girls' wellbeing in post-conflict Sri Lanka established an elevated prevalence (31%) of girl child marriage in conflict areas [30]. That same study also collected qualitative data which suggested that the rate of girl child marriage was driven by conflict-related factors such as fear of children's recruitment into militant factions [30]. This conflict-related strategy of caregivers to "protect" girl children by marrying them has been found in other conflict-affected settings as well, including among Syrian and Sudanese refugee families exposed to conflict [21, 22]. Our findings that suggest girl child marriage partially mediates the increased likelihood of IPV experience among women exposed to conflict are supported by studies from post-conflict Uganda and the Democratic Republic of Congo, where girl child marriage was associated with greater likelihood of IPV [18, 46]. While there are other drivers responsible for a major part of the increased odds of IPV among women living in areas central to conflict, girl child marriage does appear to partially mediate and explain this relationship in Sri Lanka, supporting qualitative claims of its differential practice in conflict and post-conflict areas in Sri Lanka and other research on its association with IPV.

Although our study confirmed that living in areas central to conflict in Sri Lanka is associated with increased IPV and girl child marriage, we found very different relationships in the proximal districts which bordered the conflict zone. Women living in proximal districts had a significantly lower odds of physical and emotional, and marginally significant (*p* = 0.1) lower odds of sexual IPV compared to women in areas distal to conflict. Additionally, there was no significant difference in the odds of girl child marriage in proximal districts compared to distal districts. Other studies on the Sri Lankan conflict suggest some potential explanations for the counterintuitive seeming “protective” effect on IPV among women bordering the conflict areas. One study found increased health utilization in districts bordering the conflict zones, and suggested that people who were able to leave the conflict areas might have done so to access needed health services [47]. This idea of differential mobility, that in wartime some are able to leave and others are not, was reiterated in a 2020 report on collective violence in Sri Lanka [48]. Although the author’s focus was on individuals and families able to emigrate from Sri Lanka during wartime, the idea can also be applied to individuals within the country who were able to move outside of the war zone but chose to remain nearby, in the proximal districts. These individuals might have been less at risk for IPV based on structural factors that also allowed them to escape the conflict zones. Men who remained in the conflict areas might be impacted by a larger number of risk factors for post-conflict IPV perpetration such as war-related trauma [12, 13, 49], alcohol use [10, 11], changing gender norms and roles [14], and conflict- and displacement-related economic stressors including poverty and unemployment [10, 14]. In contrast, those who were able to move to the proximal areas might have experienced fewer stressors as a result. Additionally, it is possible that these areas closest to the conflict areas have developed greater services and resources than in distal areas based on the need to provide for those fleeing conflict. Researchers in other post-conflict settings have highlighted the role that structural factors can play in sustaining IPV beyond the period of armed violence [7, 8]. It is possible that existing protective factors in the proximal areas where IPV is less likely could be expanded into both conflict-affected and distal areas in order to reduce IPV experience in those areas.

### Limitations

This study had multiple limitations. All responses were collected at the same time, so the relationships we are assessing are only cross-sectional associations, and we cannot infer causality from the findings. Our study is focused on whether child marriage is a mediator of the relationship between proximity to conflict and IPV and we have framed our analyses to address this question. Other researchers with different research questions about the role of variables such as education or parity in the relationship between proximity to conflict and IPV could reproduce our analyses with those variables in the role of mediator. The statistical techniques we used to assess mediation can also be used to identify statistical confounding, and it is possible that the observed relationship in our analyses is one of confounding rather than mediation [50]. Future studies to identify and measure mediation effects could incorporate causal inference techniques in the form of directed acyclic graphs or path diagrams to measure the relationships between the variables of interest. [51].

A small proportion of women reported being married as children, which may have underpowered our analyses to identify significant differences in girl child marriage between women in areas proximal to conflict and those in areas distal to conflict. It is possible that women responding to the survey may have underreported IPV or girl child marriage due to social desirability bias, or that women from districts with differing proximity to conflict recalled IPV experiences differently due to their varied contexts. The DHS questionnaire has been developed and tested in many contexts, including post-conflict settings, and the survey questions have been developed to reduce bias in responses.^52^ The survey did not collect complete information on women's location over time, only collecting information on the most recent location that women had moved from. Therefore, we cannot make conclusions about the direct impact of the conflict on survey participants, as it may have varied greatly based on their location over time. For this reason, our "proximity to conflict" variable must be interpreted in a post-conflict and cross-sectional context, seven years after the end of the Sri Lankan civil war, and as a geographic, rather than experiential, variable. In our partial mediation models, the 95% confidence intervals for the compared adjusted odds ratios overlap, suggesting that the observed mediation effect could possibly be due to chance. This concern is mitigated by the observation of repeated decreased odds ratios and partial mediation among all types of IPV that were studied.

## Conclusions

In this study, we found that women in areas central to conflict in Sri Lanka had increased odds of experiencing sexual, physical, and emotional IPV compared to women in areas that were distal to conflict. We also found that centrality to conflict was associated with increased odds of girl child marriage, and that girl child marriage partially mediated the association between centrality to conflict and increased odds of IPV. Additionally, we found that women in areas proximal to conflict were less likely to experience IPV and not significantly more likely to experience girl child marriage than women in areas distal to conflict. These findings highlight the long-lasting impact of the Sri Lankan conflict on women and girls' wellbeing and suggest as a possible solution that protective factors and programs that are successfully preventing IPV and girl child marriage in areas bordering the conflict should be expanded into areas central to the conflict to support women and girls living there.

## Supplementary Information

**Additional file 1.** District of residence of currently partnered women age 18-49 who participated in the 2016 Sri Lanka DHS Domestic Violence module and distribution of past year sexual, physical, and emotional intimate partner violence (IPV) (N = 13,691).

## Data Availability

Data from the 2016 Sri Lankan Demographic and Health Survey can be requested from the Sri Lankan Department of Census and Statistics. Information on requesting this data can be found at the Department's website: http://www.statistics.gov.lk/.

## Abbreviations

aOR: Adjusted odds ratio
CI: Confidence interval
DHS: Demographic and health survey
IPV: Intimate partner violence
OR: Odds ratio
SMAM: Singulate mean age of marriage
