# Supplementary material for: A mediation analysis of the role of girl child marriage in the relationship between proximity to conflict and past-year intimate partner violence in post-conflict Sri Lanka
Source: Confl Health. 2022 Feb 14;16:5. doi: 10.1186/s13031-022-00436-2 (PMC8842814; doi:10.1186/s13031-022-00436-2)
Supplement: Supplementary file 1 — Additional file 1. District of residence of currently partnered women age 18-49 who participated in the 2016 Sri Lanka DHS Domestic Violence module and distribution of past year sexual, physical, and emotional intimate partner violence (IPV) (N = 13,691). [file 13031_2022_436_MOESM1_ESM.pdf]

XIII. Additional File

Additional File 1. District of residence of currently partnered women age 18-49 who participated in the 2016 Sri Lanka DHS Domestic Violence module and distribution of past year sexual, physical, and emotional intimate partner violence (IPV) (N=13,691)

	Total		Past year sexual IPV			Past year physical IPV			Past year emotional IPV		
**District**	**n**	**%^**	**n**	**%**	**Chi-squared p-value**	**n**	**%**	**Chi-squared p-value**	**n**	**%**	**Chi-squared p-value**
Distal to conflict											
**Colombo**	1069	10%	16	1%	<0.01*	76	7%	<0.01*	124	11%	<0.01*
**Galle**	640	5%	10	2%		45	7%		119	19%	
**Gampaha**	1101	10%	14	1%		103	10%		66	6%	
**Kalutara**	603	6%	12	2%		31	5%		34	5%	
**Kandy**	808	7%	17	2%		54	6%		188	24%	
**Kegalle**	492	4%	2	0%		18	4%		34	7%	
**Kurunegala**	1034	9%	23	2%		75	7%		69	7%	
**Matara**	550	4%	0	0%		14	3%		88	15%	
**Nuwaraeliya**	480	3%	8	2%		52	11%		53	12%	
**Ratnapura**	826	6%	8	1%		61	8%		92	12%	
Proximal to conflict											
**Anuradhapura**	590	5%	10	2%		27	6%		34	6%	
**Badulla**	506	4%	6	1%		40	8%		43	8%	
**Hambantota**	442	3%	4	1%		22	5%		13	3%	
**Matale**	304	2%	21	5%		49	16%		76	26%	
**Polonnaruwa**	343	2%	3	1%		20	6%		23	7%	
**Puttalam**	535	4%	13	2%		67	12%		55	10%	
Central to conflict											
**Ampara**	578	4%	41	7%		56	10%		128	22%	
**Batticaloa**	416	3%	37	9%		122	29%		177	43%	
**Jaffna**	374	3%	26	7%		80	22%		129	36%	
**Kilinochchi**	283	1%	16	6%		66	23%		134	45%	
**Mannar**	343	0%	20	6%		54	17%		50	15%	
**Mullaitivu**	271	0%	8	3%		36	14%		78	30%	
**Trincomalee**	343	2%	37	11%		78	24%		73	22%	
**Vavuniya**	349	1%	8	2%		22	7%		52	16%	

n values are unweighted, while percent values are weighted
Chi-squared p-values are weighted according to the survey's complex sampling design
^ Total percent values are calculated within the same column, while all other percent values are calculated across the same row
**p<0.05*
